# Reactivation and Foetal Infection in Pregnant Heifers Infected with *Neospora caninum* Live Tachyzoites at Prepubertal Age

**DOI:** 10.3390/vaccines10081175

**Published:** 2022-07-25

**Authors:** Yanina P. Hecker, Mercedes M. Burucúa, Franco Fiorani, Jaime E. Maldonado Rivera, Karina M. Cirone, Matías A. Dorsch, Felipe A. Cheuquepán, Lucía M. Campero, Germán J. Cantón, Maia S. Marín, Luis M. Ortega-Mora, Dadín P. Moore

**Affiliations:** 1 Instituto de Innovación para la Producción Agropecuaria y el Desarrollo Sostenible (IPADS), Instituto Nacional de Tecnología Agropecuaria (INTA)—Consejo Nacional de Investigaciones Científicas y Técnicas (CONICET), Balcarce 7620, Argentina; burucua.mercedes@inta.gob.ar (M.M.B.); fiorani.franco@inta.gob.ar (F.F.); cirone.karina@inta.gob.ar (K.M.C.); cheuquepan.felipe@inta.gob.ar (F.A.C.); campero.lucia@inta.gob.ar (L.M.C.); canton.german@inta.gob.ar (G.J.C.); marin.maia@inta.gob.ar (M.S.M.); moore.dadin@inta.gob.ar (D.P.M.); 2 Facultad de Ciencias Agrarias, Universidad Nacional de Mar del Plata, Balcarce 7620, Argentina; jaime.maldonado@ucuenca.edu.ec (J.E.M.R.); matiasdorsch@gmail.com (M.A.D.); 3 Facultad de Ciencias Agropecuarias, Universidad de Cuenca, Cuenca 010205, Ecuador; 4 Plataforma de Investigación en Salud Animal, Instituto Nacional de Investigación Agropecuaria (INIA), Estación Experimental La Estanzuela, Colonia 70000, Uruguay; 5 SALUVET, Departamento de Sanidad Animal, Facultad de Ciencias Veterinarias, Universidad Complutense de Madrid, 28040 Madrid, Spain; luis.ortega@ucm.es

**Keywords:** *Neospora caninum*, bovine, prepubertal inoculation, live parasite, reactivation, safety, efficacy, vertical transmission

## Abstract

*Neospora caninum* is recognised for causing cattle abortion, provoking severe economic losses in the livestock industry worldwide. The aim of the present study was to evaluate the reactivation and foetal infection in pregnant heifers inoculated with live *N. caninum* tachyzoites before puberty. A total of 15 30-month-old pregnant heifers were allocated into four groups: animals inoculated with live tachyzoites of NC-Argentina LP1 isolate before puberty and challenged with live tachyzoites of NC-1 strain at 210 days of gestation (DG) (Group A); animals mock inoculated before puberty and challenged with NC-1 strain at 210 DG (Group B), animals inoculated before puberty but not subsequently challenged (Group C); and noninfected and nonchallenged animals (Group D). The results of this study showed that 100% of animals infected before puberty (Groups A and C) suffered reactivation of the infection at the seventh month of gestation. In addition, in three and two calves from Groups A and C, respectively, congenital infection was confirmed. Interestingly, we provide evidence that the use of live *N. caninum* tachyzoites in young animals as a strategy to induce protection is neither safe nor effective.

## 1. Introduction

*Neospora caninum* is an apicomplexan protozoan parasite recognised for causing reproductive failure in cattle, provoking severe economic losses in the livestock industry worldwide [1]. Bovines can be infected horizontally by the ingestion of *N. caninum* oocysts shed in the faeces of definitive hosts (canids). However, the transplacental route (vertical transmission) from dam to foetus is the most efficient mode of transmission for this protozoan parasite [2]. Bovine neosporosis is highly disseminated around the world, reaching up to 90% seroprevalences on some farms [2]. Abortion is the main clinical sign, with *N. caninum*-infected cows having an increased risk of repetitive abortions in consecutive pregnancies [1]. Although more than one billion dollars per year are lost due to bovine neosporosis, the control measures are limited to herd management because neither effective vaccines nor drugs are currently available against this parasite [1,3,4].

In bovines, the protective immune response against *N. caninum* is associated with cytotoxic T cells and the production of cytokines such as interferon-gamma (IFN-γ), interleukin-12 (IL-12) and tumour necrosis factor-alpha (TNF-α) [5,6,7]. On the other hand, the humoral immune response is only protective during parasitaemia [7]. The presence of specific *N. caninum* antibodies is indicative of parasite exposure and is associated with a higher risk of vertical transmission and abortions [2]. Field observations suggested that naturally exposed cattle develop protective immune mechanisms against abortions in a subsequent *Neospora*-related outbreak [8]. Furthermore, endogenous transplacental transmission is more likely to occur in cattle than post-natal infection [2,4,9].

It has been reported that *N. caninum* vertical transmission could be prevented when cows are experimentally inoculated with live tachyzoites before mating and then challenged during their gestation [9]. For this reason, live vaccines based on attenuated or low virulent parasites have been considered the most promising prophylactic measure. However, the use of live parasites as an immunogen has disadvantages because some animals may become chronically infected and transmit *N. caninum* to their progeny [4]. Recently, our group showed that inoculation with live tachyzoites of the NC-Argentina LP1 local isolate [10] in 6-month-old female calves elicited a specific cellular immune response with antibody levels that decreased at Day 120 post-infection (PI) [11]. Nevertheless, whether these infected animals could reactivate the infection and transmit the parasite during their reproductive life on the farm was not studied. In addition, it would be interesting to know if the memory immune response generated at a young age in these animals could protect against a heterologous challenge. Therefore, the aim of the present study was to evaluate the reactivation and foetal infection in pregnant heifers inoculated with live *N. caninum* tachyzoites before puberty.

## 2. Materials and Methods

### 2.1. Animals and Experimental Samplings

**Figure 1:**
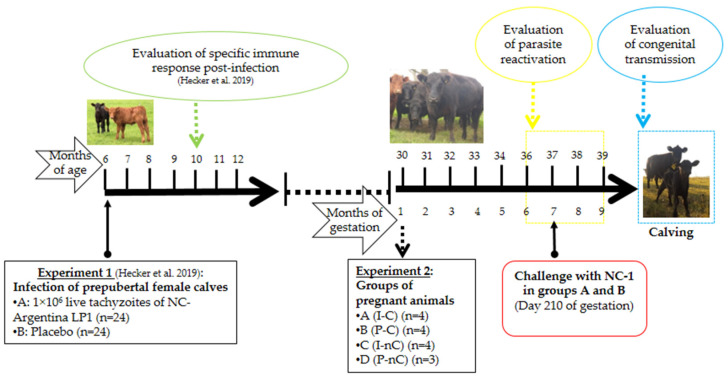
Chronological layout of the experiment. A chronological summary of Experiments 1 and 2 is depicted. Experiment 1 involved the inoculation of prepubertal female calves with the *Neospora* NC-Argentina LP1 isolate (already described in [11]), while Experiment 2 involved the results included in the present study.

Fifteen 30-month-old pregnant Angus heifers from a beef herd located at INTA Balcarce, Argentina, which were involved in a previous study (Figure 1) [11], were allocated into four groups (Table 1): four animals that had been inoculated subcutaneously (SC) with 1 × 10^6^ live tachyzoites of the NC-Argentina LP1 isolate before puberty (at 6 months old) [11] and were later challenged intravenously (IV) with 1 × 10^8^ live tachyzoites of the NC-1 strain at 210 days of gestation (DG) (Group A(I-C)); four animals that had been mock inoculated with phosphate buffered saline (PBS, pH 7.2) before puberty and subsequently challenged IV with 1 × 10^8^ live NC-1 tachyzoites at 210 DG (Group B(P-C)), four animals that had been inoculated SC with 1 × 10^6^ live tachyzoites of NC-Argentina LP1 strain before puberty (at 6 months old) [11] and were not challenged (Group C(I-nC)); and three animals that had been mock inoculated with PBS before puberty and not challenged (Group D(P-nC)). All animals were fed on a natural pasture in one paddock and maintained under standard animal husbandry conditions. Clean water was always available.

**Table 1:** Study design and groups. ^(1)^ BP: before reaching puberty; ^(2)^ DG: days of gestation.

Group (*n*)	Treatment BP ^(1)^	Challenged at 210 DG ^(2)^ with NC-1	Evaluation of
A (4)	1 × 10^6^ live tachyzoites of NC-Argentina LP1 strain	Yes	Protection
B (4)	PBS	Yes	Challenge control
C (4)	1 × 10^6^ live tachyzoites of NC-Argentina LP1 strain	No	Reactivation
D (3)	PBS	No	Negative control

During gestation, blood samples from dams (40 mL) were collected by jugular puncture in tubes without sodium citrate buffer (pH 6.2) and centrifuged at 1600× *g* for 10 min, and sera were obtained and stored at −20 °C until use. To determine the total IgG levels throughout gestation, an in-house indirect enzyme-linked immunosorbent assay (iELISA) and indirect immunofluorescence antibody test (IFAT) were performed monthly. In addition, to evaluate parasite reactivation at the sixth, seventh, eighth and ninth month of gestations, antibody detection was complemented with an immunoblot (IB) assay. Moreover, blood samples collected in tubes with sodium citrate buffer were obtained and immediately processed to obtain peripheral blood mononuclear cells (PBMCs) at the sixth, seventh, eighth, and ninth months of gestation. PBMCs were isolated by centrifugation on Ficoll-Paque™ PLUS (density: 1.077 g/mL; GE Healthcare Bio-Sciences AB, Uppsala, Sweden) following the manufacturer’s recommendations. Cells were counted under the microscope after staining with Trypan blue. Then, DNA detection was performed on PBMCs by nested PCR (see below). To analyse the cellular immune response in the last 4 months of gestation, cytokine expression levels were also measured in these PBMCs.

At calving, jugular blood samples were collected from dams and their calves before colostrum intake. Similarly, colostrum and placenta samples were also obtained soon after parturition. Blood samples from calves without anticoagulant and colostrum samples were processed following the same procedure described above to obtain serum. To ensure the absence of colostrum intake before sampling in calves, gamma-glutamyl transferase (GGT) activity was measured in sera using the γ-G-test (Wiener lab, Rosario, Argentina) according to the manufacturer’s instructions, where GGT values above 50 IU/L were indicative of colostrum intake [12]. To evaluate *Neospora* infection in calves, a serological study was performed by iELISA, IFAT and IB. In addition, blood samples with anticoagulants from calves were also processed to obtain PBMCs. The presence of parasite DNA was assessed in the placenta, calf PBMCs and umbilical cords by PCR.

### 2.2. Neospora Caninum Inoculum and Native Antigen Extract

The inoculation in prepubertal female calves with live tachyzoites of the NC-Argentina LP1 strain [10] and the obtaining of the native antigen extract for the IgG and its subisotypes ELISAs have been previously described [11]. Protein content was determined using the Micro BCA protein assay method (Pierce, Rockford, IL, USA), and the supernatant was aliquoted and cryopreserved at −80 °C until use.

Live tachyzoites of the NC-1 strain of *N. caninum* [13] were cultured in Vero cells to obtain the challenge inoculums as previously described [14]. Tachyzoite numbers and viability were determined by trypan blue exclusion counting using a haemocytometer. In addition, immediately after the field inoculation, an aliquot of the inoculum was harvested in Vero cells to check the viability of the challenge inoculum.

### 2.3. Specific IgG and Its Subisotypes in Serum and Colostrum Samples

*Neospora caninum*-specific IgG levels were determined by an in-house iELISA on serum samples from dams and calves (1:100 dilution) following the methodology previously described by Hecker et al. [11]. An anti-bovine IgG polyclonal antibody conjugated to peroxidase (1:1000; Sigma Chemical Co., St. Louis, MO, USA) was used as the secondary antibody. *Neospora caninum*-specific IgG levels were evaluated using an Epoch microvolume spectrophotometer system (Epoc, Bioteck^®^ Instruments, Inc., Winooski, VT, USA) at an optical density (OD) of 405 nm (OD_405_) when the *N. caninum* positive control reached a 1.0 ± 25% OD_405_ value. Samples were analysed in duplicate, and the OD mean value was converted into a relative index percent (RIPC) using the following formula: RIPC = (OD_405_ sample − OD_405_ negative control)/(OD_405_ positive control − OD_405_ negative control) × 100. A RIPC value ≥ 8.2 was considered a positive result [14].

Colostrum samples obtained immediately after calving were assessed for specific IgG antibodies using a commercial ELISA kit (CIVTEST™, Hipra, Amer, Spain). Colostrum samples were also analysed in duplicate, and RIPC values higher than 10 indicated a positive serological status [15].

*Neospora caninum*-specific IgG1 and IgG2 subisotypes were determined by iELISA on maternal serum samples at months 6, 7, 8, and 9 of gestation, following the methodology previously described by Maldonado et al. [15]. The anti-bovine IgG1 and IgG2 monoclonal antibodies (provided by Dr. S. Srikumaran, University of Nebraska, Lincoln, NE, USA) were used. Absorbance was recorded at 492 nm, and a cut-off was established as the mean A492 of the negative sera +2 standard deviations (SDs).

### 2.4. Indirect Immunofluorescence Antibody Test (IFAT)

Detection of specific *N. caninum* antibodies was carried out by IFAT as previously described [16]. Briefly, suspensions of *N. caninum* (NC-1) tachyzoites (5 × 10^6^ mL^−1^) in PBS were air dried on glass slides, fixed with ice-cold acetone for 5 min, and then used as antigens [12]. A fluorescein isothiocyanate-labelled affinity-purified rabbit anti-bovine IgG antibody (Sigma) was used, and a cut-off dilution of 1:25 was defined for dam and calf serum [16]. The highest serological dilution with complete peripheral positive reactions was considered the endpoint titre. Positive control sera were obtained from *N. caninum* experimentally infected cows [14]. Slides were examined with an epifluorescence microscope (Olympus Bx 51, Olympus Inc., Tokyo, Japan). Antibody titres were expressed as the reciprocal of the highest serum dilution that showed complete peripheral fluorescence of tachyzoites [12].

### 2.5. Immunoblot (IB) Analysis

Detection of specific *N. caninum* antibodies was carried out by IB following the methodology previously described by Campero et al. [17]. Briefly, we used native antigen extracts under non-reducing conditions and dam and calf serum samples were diluted 1:100. The reaction against five immunodominant antigens (IDAs) with relative molecular masses of 19, 29, 30, 33, and 37 kDa was recorded in the IB from crude tachyzoite antigen. Samples were considered positive when two or more IDAs were recognised.

### 2.6. Nested PCR (nPCR) for Neospora Caninum

DNA was extracted from PBMCs (from heifers and calves), placenta, colostrum, umbilical cord, and aborted foetus tissues using a commercially available kit (High Pure PCR Template Preparation Kit, Roche, Mannheim, Germany) following the manufacturer’s recommendations. The DNA concentration was measured using the Epoch microvolume spectrophotometer system, and the samples were diluted to a final concentration of 60 ng/μL. For molecular detection, nested PCR targeting the internal transcribed spacer 1 (ITS-1) region of *N. caninum* was carried out with four oligonucleotides as described by Buxton et al. [18]. Secondary amplification products were visualised by electrophoresis in 2% agarose gels and SYBR Safe staining. DNA equivalent to 10^2^ tachyzoites was used as the positive PCR control. Negative controls (molecular grade water) were included for each set of DNA extractions and PCRs.

### 2.7. Histopathological Studies on Placentas

Placentomes or intercotyledonary chorion region samples taken at delivery, as well as foetal tissue samples from the aborted foetus, were fixed in 10% buffered formalin, paraffin embedded, cut into 5 µm sections, mounted on glass microscope slides, stained with haematoxylin and eosin (H & E), and examined by microscopy (Olympus, Tokyo, Japan) following standard methods described by Campero et al. [19].

### 2.8. Cytokine mRNA Expression in Dam PBMCs

Total RNA from PBMCs from dams and calves was extracted using TRIzol reagent (Invitrogen, Carlsbad, CA, USA) according to the manufacturer’s protocol. RNA was digested with DNase I Amplification Grade (Invitrogen) for 30 min at 37 °C to remove any contaminating genomic DNA (gDNA). The quality and quantity of the resulting RNA were determined using the Epoch microvolume spectrophotometer system. All RNA samples were stored at −80 °C until use. Complementary DNA (cDNA) was synthesised using a reaction mixture containing 1 μg of total RNA, random hexamers (12 ng/μL) (Promega, Madison, WI, USA), and Moloney murine leukaemia virus reverse transcriptase (10 U/μL) (Promega, Madison, WI, USA). Negative controls, omitting the RNA or reverse transcriptase, were included.

Real-time PCR was performed using primers targeting the bovine interleukin-10 (IL-10), interferon-γ (IFN-γ), tumour necrosis factor-α (TNF-α), interleukin-12 p40 (IL-12 p40), interleukin-4 (IL-4), and the housekeeping glyceraldehyde-3-phosphate dehydrogenase (GAPDH) genes on cDNA samples from PBMCs collected at the sixth, seventh, eighth, and ninth month of gestation [15,20]. The amplification efficiency was determined for each gene using 10-fold dilutions of the cDNA. Relative quantification of cytokine mRNA expression levels was carried out using the 2^−ΔΔCT^ method [11,21]. PBMCs from Group D were used as negative controls.

### 2.9. Statistical Analysis

The IgG and cytokine RNA levels in animals from different groups throughout the experiment were analysed by a two-way ANOVA repeated measures model followed by Tukey’s multiple comparisons test. All analyses were performed using the GraphPad Prism 8.0.1 program (San Diego, CA, USA), and differences among groups were considered statistically significant when *p* ≤ 0.05. The RT–qPCR efficiency for each gene was determined by a linear regression model according to Equation = 10 (−1/slope).

## 3. Results

Neither apparent clinical signs nor significant local reactions were observed in any heifers from Groups A and B after challenge at 210 DG. Challenged animals did not develop fever between 24 and 120 h PI (data not shown). The viability of the inoculum used in the field trial was confirmed, as lysis plaques were observed in Vero cell cultures 48 h after inoculation.

At delivery, 14 out of 15 heifers involved in this study gave birth to clinically healthy calves, and none of the dams exhibited placental retention. One heifer from Group A (#12) aborted 35 days post-challenge, although the diagnosis of *N. caninum* in the aborted foetus could not be confirmed (see below). All calf serum samples showed GGT values below 50 IU/L, confirming that no colostrum intake occurred before sampling.

### 3.1. Serum Antibody Response in Dams

#### 3.1.1. Specific Total IgG

**Figure 2:**
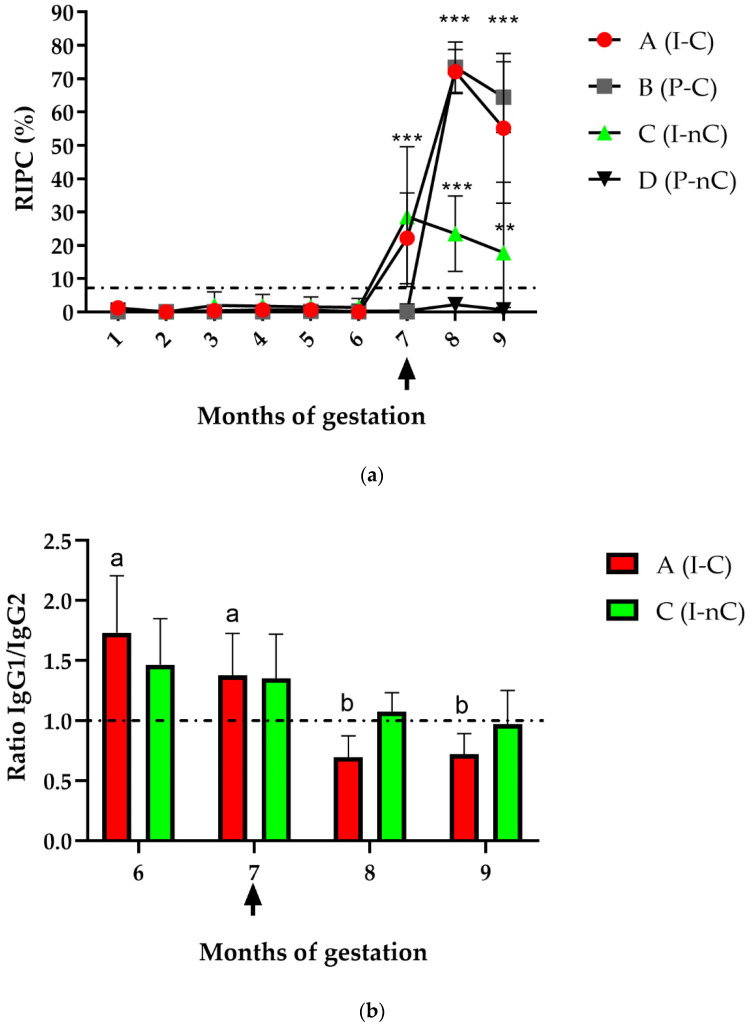
Longitudinal profile of immunoglobulin G (IgG) and subisotypes in heifers from different experimental groups assessed by iELISA. Each dot represents the mean ± standard deviation (SD) at different sampling times. (**a**) Mean ± SD of the relative index percentages (RIPC) ± SD of total IgG throughout gestation in Groups A (inoculated and challenged (I-C)), B (placebo and challenged (P-C)), C (inoculated and not challenged (I-nC)), and D (placebo and not challenged (P-nC)). Cut-off: ≥8.2 RIPC. *** *p* < 0.0001. ** *p* < 0.001. The upwards arrow marks the day of challenge (210 days of gestation). (**b**) Mean *Neospora*-specific IgG1/IgG2 ratio ± SD of animals in Groups A and C at 6, 7, 8, and 9 months of gestation. Lowercase letters indicate significant differences within the same group over time. The upwards arrow marks the day of challenge (210 days of gestation).

The RIPC values detected by iELISA in dams from all groups during gestation (before and after challenge) are shown in Figure 2a. From the first until the sixth month of gestation, no specific IgG levels were detected in any of the animals by iELISA. At the seventh month of gestation, an increase in the antibody levels of heifers from Groups A (I-C) and C (I-nC) was detected, which were significantly different from those in Groups B (P-C) and D (P-nC) (*p* < 0.0001). After NC-1 challenge (eighth and ninth month of gestation), RIPC values increased significantly in animals from Groups A and B compared to Groups C and D (*p* < 0.0001). Additionally, heifers from Group C remained seropositive until the ninth month of gestation, remaining significantly different from Group D (*p* < 0.005).

A summary of the antibody detection in dam sera at months 6, 7, 8, and 9 of gestation by iELISA, IFAT, and IB is shown in Table 2. All heifers from Groups A and C (animals inoculated before puberty) were seropositive by IFAT (titre > 1:25) from the first until the sixth month of gestation, with antibody titres lower than 1:50 (data not shown). From the seventh month until delivery, the antibody levels increased significantly in both groups compared with Groups B and D, with different endpoint titres (Table 2). Moreover, Group A heifers significantly increased their antibody levels at the eighth month of gestation (after challenge) compared with Group C heifers (*p* < 0.0001). In addition, animals from Group B were IFAT positive at the eighth month (after challenge) with endpoint titres > 1:6400, exhibiting a statistically significant increase in antibodies when compared to Groups C and D (*p* < 0.0001). Finally, fluctuating antibody levels were detected by IB (using a cut-off of 1:100) in Groups A and C from the sixth month until delivery (Table 2). After challenge, animals from Group B were positive by IB, recognising two or more IDAs in all samples from this group.

At delivery, colostrum samples showed specific IgG antibodies against *N. caninum* in three of the four dams from Group A and in two of the four dams from Group C (Table 3). In Group B, specific antibodies against *N. caninum* were only detected in one colostrum sample (#32). Finally, all colostrum samples from Group D were seronegative.

#### 3.1.2. IgG Subisotypes

The *N. caninum*-specific IgG1/IgG2 ratios in Groups A (I-C) and C (I-nC) during months 6, 7, 8, and 9 of gestation are shown in Figure 2b. Ratios > 1 in both groups were observed during the sixth and seventh months of gestation. After challenge (eighth and ninth month of gestation), Group A heifers showed ratios < 1, but the ratio in Group C remained close to 1. There were no significant differences in the ratio between the A and C groups in any month, but a significant change in the IgG1/IgG2 ratio within Group A occurred over time (*p <* 0.005). On the other hand, Group B showed ratios < 1 during months 8 and 9 (after challenge) (data not shown). Finally, in colostrum samples, the predominant subisotype in all seropositive samples from different experimental groups (#4, #5, #9, #32, #18, and #19) was IgG1 (data not shown).

### 3.2. Cellular Immune Response in Dams

**Figure 3:**
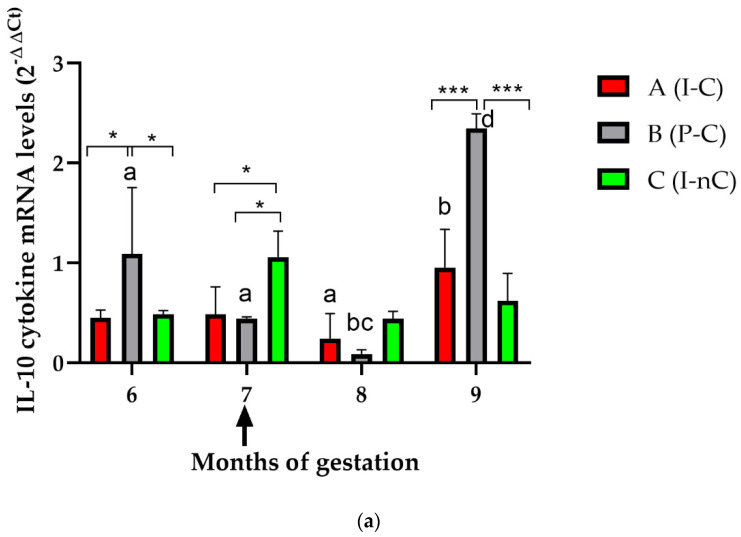

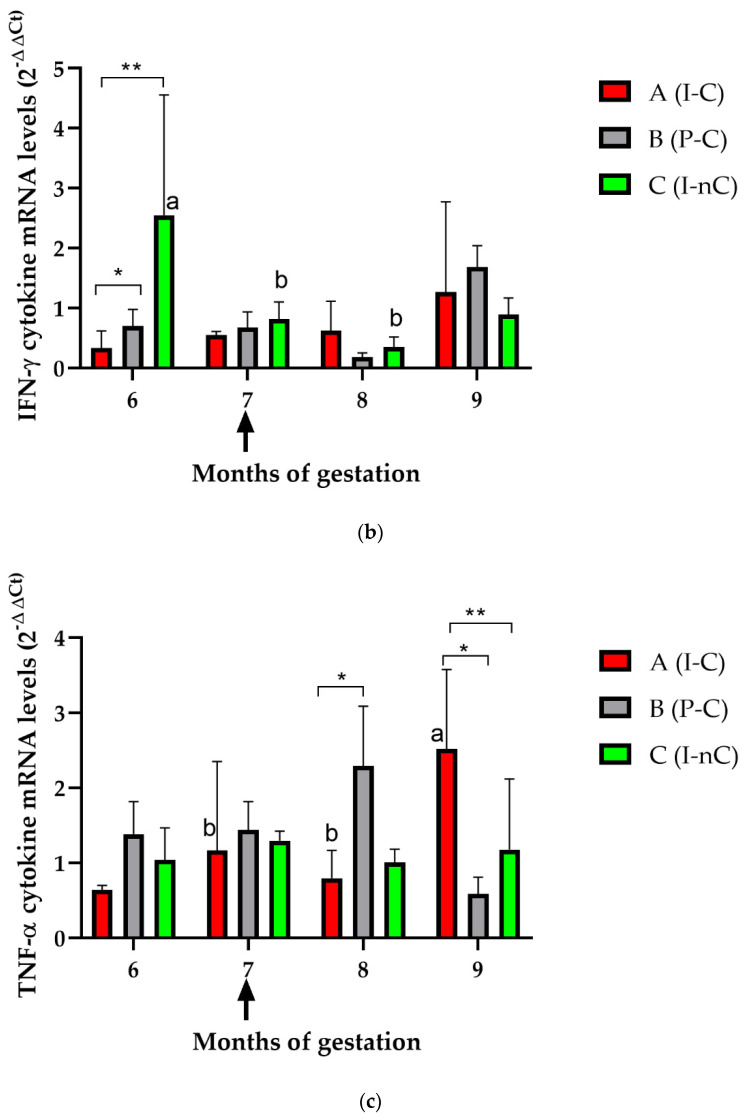

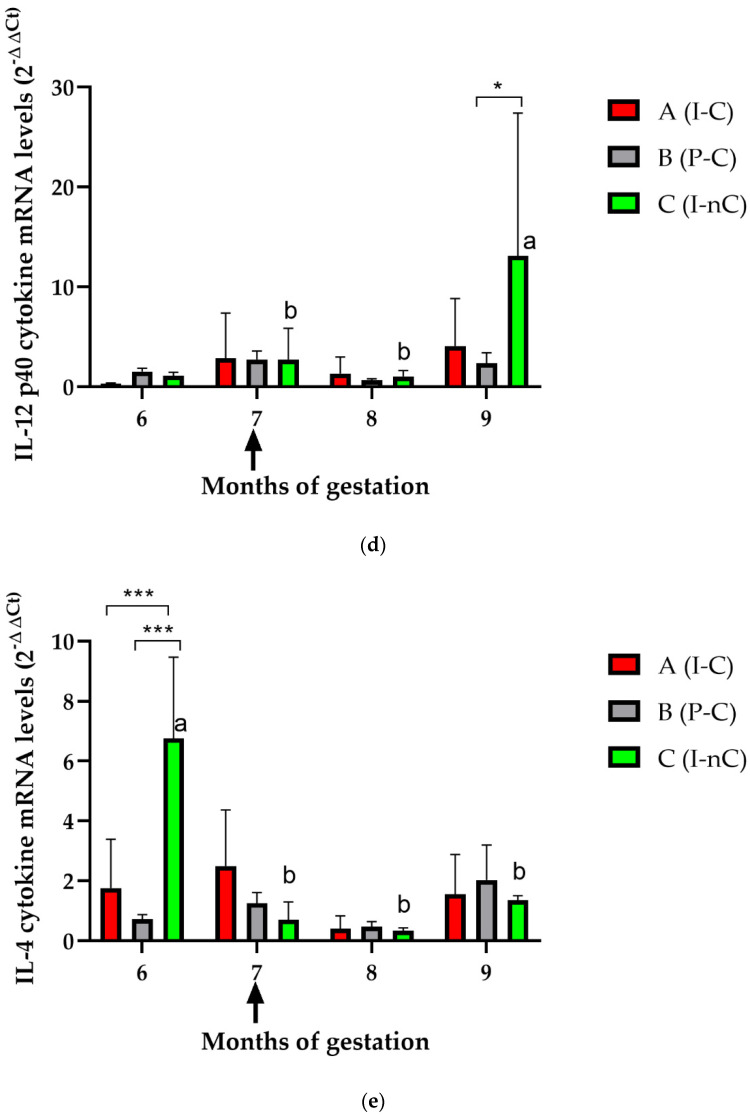
Interleaved-bar graphs of relative cytokine mRNA expression levels in PBMCs in heifers from Groups A (inoculated and challenged (I-C)), B (placebo and challenged (P-C)), and C (inoculated and not challenged (I-nC)). Relative quantification of cytokine mRNA expression levels was carried out using the 2^−ΔΔCt^ method. The numbers on the Y-axis represent the mean fold change in cytokine mRNA expression levels relative to Group D (placebo and no challenge (P-nC), i.e., the negative control). Error bars represent the SD. (**a**) IL-10; (**b**) INF-γ; (**c**) TNF-α; (**d**) IL-12 p40; and (**e**) IL-4. Upwards arrow: day of challenge (210 days of gestation). The significant differences among groups were analysed. *** *p* < 0.0001; ** *p* < 0.001; * *p* < 0.05. Letters indicate significant differences in each group over time.

The relative levels of cytokine mRNA in PBMCs from Groups A, B, and C during months 6, 7, 8, and 9 of gestation are shown in Figure 3.

At the sixth month of gestation, inoculated animals before puberty (Groups A and C) showed lower IL-10 mRNA levels (*p* < 0.05) than uninfected animals (Group B) (Figure 3a). At the seventh month, the IL-10 levels increased slightly (*p* < 0.05) in Group C (I-nC) compared with Groups A (I-C) and B (P-C). At the eighth month, there were no differences between groups. However, at the ninth month, there was a statistically significant increase in IL-10 levels after challenge in Group B compared with Groups A and C (*p* < 0.0001). Finally, there were differences in the IL-10 levels over time in Groups A and B, which showed significantly higher cytokine levels after challenge (months 8 and 9 of gestation) (*p* < 0.0001).

When the IFN-γ mRNA levels were analysed (Figure 3b), differences were only observed at the sixth month of gestation, when Group C expressed higher levels than Groups A and B (*p* < 0.05). In addition, differences over time were also observed in Group C (*p* < 0.05).

Differences among groups in TNF-α mRNA levels were observed at month 8 between Groups A and B (*p* < 0.05), at month 9 between Groups A and B (*p* < 0.05) and between Groups A and C (*p* < 0.001) (Figure 3c). In addition, differences over time were observed in Group A after challenge (*p* < 0.001).

The IL-12 p40 mRNA levels were different between Groups B and C at the ninth month (*p* < 0.05) (Figure 3d). Differences over time were only observed in Group C, which showed higher IL-12 p40 mRNA levels at month 9.

Significantly higher IL-4 mRNA levels were observed in Group C at month 6 than in Groups A and B (*p* < 0.0001) (Figure 3e). Differences over time were only found in Group C, which showed the highest levels at month 6 (*p* < 0.005).

### 3.3. Neospora DNA Detection in Dams during Gestation and at Delivery

The presence of *Neospora* DNA was analysed in PBMCs from heifers in Groups A (I-C) and C (I-nC) from the sixth month of gestation until delivery, with the aim of detecting parasite reactivation in animals that had been inoculated before puberty (Table 2). Although only one animal from Group A had detectable DNA in PBMCs (#12) at month 6 of gestation, at the seventh month, all heifers from Group A (before challenge) and Group C had *Neospora* DNA in their blood, confirming parasitaemia in these animals. At the eighth month (after challenge in Group A), only two dams from Group A (#9 and #12) and two dams from Group C (#2 and #19) had parasite DNA. Finally, at month 9 and at delivery, three animals from Group A showed *Neospora* DNA in their PBMCs (#4, #5 and #12).

When placenta and colostrum samples were analysed, the level of DNA detection was low, with only positive one placenta from Group A (#5). Finally, *N. caninum* DNA was not detected in any foetal tissues from Group A.

### 3.4. Histopathological Studies on Placentas and Aborted Foetuses

Minimal to mild inflammatory changes, possibly associated with delivery, were observed microscopically in the placentas from all groups. Microscopic lesions were limited to focal and mild lymphohistiocytic inflammatory infiltrates and the presence of small haemorrhagic and necrotic foci in intercotyledonary areas. No differences in lesion severity were observed between groups.

When different tissues from the aborted foetus (from Group A) were analysed, lesions compatible with *N. caninum* infection were not found. In addition, there were no specific lesions that could be associated with other infectious agents.

### 3.5. Detection of N. Caninum Infection in Calves

Serological and molecular findings in calves at delivery are shown in Table 3. Calves were considered seropositive when their serum samples tested positive for iELISA, IFAT or IB. In Group A (I-C), antibodies against *N. caninum* were detected for all the healthy calves (*n* = 3) by iELISA and IFAT (calf #1, #6, and #9) and in two of them also by IB (calf #6 and #9). However, the serologic analysis was negative in the aborted foetus from this group. On the other hand, in Group C (I-nC), only two calves were seropositive to *N. caninum* by iELISA and IFAT (#12 and #5). In addition, all calves born from Group B (P-C) dams were seropositive to *N. caninum* (by iELISA, IFAT and IB). Congenitally infected calves from Groups A, B, and C showed antibody titres ≥1:400, although no significant differences in the endpoint titres were found among groups.

When the umbilical cord and PBMC samples from calves were analysed by PCR, *Neospora* DNA was only detected in one sample from Group A (calf #6 from dam #5).

**Table 2:** Reactivation study in inoculated heifers in the last 4 months of gestation. I-C: inoculated and challenged; P-C: placebo and challenged; I-nC: inoculated not challenged; P-nC: placebo not challenged. PBMCs: peripheral blood mononuclear cells. IB: immunoblot. ^1^ Cut-off 1:100; ^2^ Cut-off 1:25; ^3^ Cut-off 1:100; ^4^ From PBMC. + and − indicate a positive and negative result, respectively, for each of the assays. Lowercase letters (a and b) indicate significant differences between different groups.

Groups	Months of Gestation	6				7				8				9			
	Heifers	ELISA ^1^	IFAT ^2^	IB ^3^	PCR ^4^	ELISA ^1^	IFAT ^2^	IB ^3^	PCR ^4^	ELISA ^1^	IFAT ^2^	IB ^3^	PCR ^4^	ELISA ^1^	IFAT ^2^	IB ^3^	PCR ^4^
A	#4	−	25 ^a^	+	−	+	100 ^a^	+	+	+	12,800 ^a^	+	−	+	6400 ^b^	+	+
(I-C)	#5	−	100 ^a^	+	−	+	800 ^a^	+	+	+	25,600 ^a^	+	−	+	6400 ^b^	+	+
	#9	−	25 ^a^	−	−	−	100 ^a^	−	+	+	25,600 ^a^	+	+	+	12,800 ^b^	+	−
	#12	−	100 ^a^	+	+	+	1600 ^a^	+	+	+	12,800 ^a^	+	+	+	800 ^b^	+	+
B	#32	−	− ^b^	−	−	−	− ^b^	−	−	+	12,800 ^a^	+	−	+	51,200 ^a^	+	−
(P-C)	#34	−	− ^b^	−	−	−	− ^b^	−	−	+	12,800 ^a^	+	−	+	25,600 ^a^	+	−
	#38	−	− ^b^	−	−	−	− ^b^	−	−	+	25,600 ^a^	+	−	+	6400 ^a^	+	−
	#47	−	− ^b^	−	−	−	− ^b^	−	−	+	25,600 ^a^	+	−	+	6400 ^a^	+	−
C	#2	−	100 ^a^	+	−	+	400 ^a^	+	+	+	800 ^b^	+	+	+	1600 ^b^	−	−
(I-nC)	#14	−	25 ^a^	−	−	+	400 ^a^	+	+	+	200 ^b^	+	−	+	200 ^b^	+	−
	#18	−	25 ^a^	+	−	−	100 ^a^	+	+	+	3200 ^b^	+	−	+	3200 ^b^	−	−
	#19	−	50 ^a^	+	−	+	400 ^a^	+	+	+	800 ^b^	+	+	+	3200 ^b^	+	−
D	#30	−	− ^b^	−	−	−	− ^b^	−	−	−	− ^c^	−	−	−	− ^c^	−	−
(P-nC)	#47a	−	− ^b^	−	−	−	− ^b^	−	−	−	− ^c^	−	−	−	− ^c^	−	−
	#48	−	^− b^	−	−	−	− ^b^	−	−	−	− ^c^	−	−	−	− ^c^	−	−

**Table 3:** *N. caninum*-serological status in dams and their offspring from different groups at delivery, before colostrum intake. I-C: inoculated and challenged; P-C: placebo and challenged; I-nC: inoculated not challenged; P-nC: placebo not challenged. PBMC: peripheral blood mononuclear cells. ^1^ Antibody detection by either iELISA, IFAT, or IB; ^2^ Cut-off 1:100; ^3^ Cut-off 1:25. + and − indicate a positive and negative result, respectively, for each of the assays.

Experimental Groups	Dams						Calves			
	ID	Specific IgG ^1^		*N. caninum* DNA			ID	Specific IgG ^1^	*N. caninum* DNA	
		Serum ^2^	Colostrum ^1^	Placenta	PBMC	Colostrum		Serum ^3^	Umbilical Cord	PBMC
A (I-C)	#4	+	+	−	+	−	#1	+	−	−
	#5	+	+	+	+	−	#6	+	+	−
	#9	+	+	−	−	−	#9	+	−	−
	#12	+	−	−	+	−	Aborted foetus	−	−	−
B (P-C)	#32	+	+	−	−	−	#3	+	−	−
	#34	+	−	−	−	−	#4	+	−	−
	#38	+	−	−	−	−	#14	+	−	−
	#47	+	−	−	−	−	#13	+	−	−
C (I-nC)	#2	+	−	−	−	−	#11	−	−	−
	#14	+	−	−	−	−	#12	+	−	−
	#18	+	+	−	−	−	#5	+	−	−
	#19	+	+	−	−	−	#10	−	−	−
D (P-nC)	#30	−	−	−	−	−	#8	−	−	−
	#47a	−	−	−	−	−	#15	−	−	−
	#48	−	−	−	−	−	#2	−	−	−

## 4. Discussion

The clinical outcome of bovine neosporosis can vary depending on whether the infection takes place prior to or after birth. Previous studies reported that endogenous transplacental transmission is more likely to occur in cattle than post-natal infection, probably due to the development of incomplete immunity when the infection occurs during gestation and because postnatally infected animals could clear the infection and develop immunity [4,22]. Innes et al. [9] reported that the experimental inoculation of naïve cows with live *N. caninum* tachyzoites 6 weeks prior to mating protected against vertical transmission following a challenge at mid-gestation. Later, Williams et al. [23] described that in chronically infected dams, the immune response protected against abortion after an exogenous challenge, but it was ineffective against reactivation of latent infection. Nevertheless, other authors have suggested that early exposure of young cattle to *N. caninum* could lead to the development of protective immunity against postnatal transmission [24,25,26]. Interestingly, the results of the present study clearly show that animals inoculated before puberty [11] had parasitic reactivation, as *Neospora*-DNA was detected in their PBMCs, and an increase in the specific antibody titres from the seventh month of gestation onwards was observed. Additionally, it has been previously mentioned that the detection of circulating parasites in the blood of infected animals is difficult, mainly because of the low number of circulating parasites and because there is a short period of parasitaemia [27,28]. In the present study, parasitaemia was confirmed in all infected females (Groups A and C) at the seventh month of gestation. This is in agreement with previous reports where it was shown that an increase in the levels of specific antibodies around mid-to-late gestation can be an indicator of parasite reactivation and proliferation [27,29,30,31]. Therefore, the novelty of the present study lies in the fact that, for the first time, we show that the inoculation of live tachyzoites of *N. caninum* in prepubertal female calves, despite inducing a specific immune response, neither prevented the reactivation of the parasite during pregnancy nor protected against heterologous challenge, evidencing that these postnatally infected animals were unable to eliminate the parasite.

At present, the development of an effective vaccine against neosporosis is one of the major challenges in the field. An alternative approach that has been evaluated by several authors consists of the injection of live tachyzoites before gestation to induce protective immunity against reinfection, considering that protection conferred by the previous infection is not specific for the isolate [9,14,32,33,34,35,36]. Additionally, the role of previous and persistent infections as a source of protection has been investigated. Low-virulence *Neospora* isolates have shown variable efficacy in preventing transplacental transmission. It has been proposed that *N. caninum* strains isolated from congenitally infected and asymptomatic calves could be suitable vaccine candidates due to their limited capacity to cause abortion in cattle while protecting against vertical transmission [32,33,34,35,36,37,38]. A partial protection effect against congenital infection was achieved when the NC-Nowra strain was inoculated in heifers prior to breeding and subsequently challenged at 139 days of gestation with a heterologous strain [35]. Additionally, Rojo-Montejo et al. [34] demonstrated a degree of protection against vertical transmission after challenge at mid-gestation (Day 135) in cows that had been immunised with 10^7^ live Nc-Spain 1H tachyzoites (a low virulent strain) prior to artificial insemination. Although the results of our previous study showed that the infection in prepubertal calves with NC-Argentina LP1 local isolate elicited a specific cellular immune response [11], in the present work, we demonstrate that the infection in prepubertal female calves is neither safe nor effective because, after the heterologous challenge, both parasite reactivation and vertical transmission were confirmed. In line with our results, Mazuz et al. [36] observed a significantly lower incidence of abortion in seropositive cows vaccinated with the live vaccine isolate NcIs491 during gestation than in nonvaccinated cows, although the number of seropositive offspring remained similar in both groups. On the other hand, Rojo-Montejo et al. [34] observed that the offspring of immunised/challenged animals had significantly lower precolostral *Neospora*-specific antibody titres than calves from the nonimmunised/challenged group. Although this result is not directly comparable to our study, because we used a different isolate, infection model, and challenge time point (210 days of gestation), in our study in the congenitally infected calves from Group A (I-C), no differences in antibody levels were detected compared to infected offspring from other experimental groups, having all calf titres ≥ 400.

Experimental *N. caninum* infection after Day 210 of gestation has not been associated with abortion or severe foetal lesions [7,22,39]. Instead, infections in the last third of gestation are often related to the birth of healthy but congenitally infected calves that will be able to transmit the parasite in successive pregnancies [39,40,41,42,43]. In the present study, neither histopathological lesions nor *Neospora* DNA was found in the placentas of infected animals from Groups B and C, and only one placenta from Group A was PCR positive. Notably, either DNA in umbilical cords or PBMCs was found in their offspring. Unfortunately, it was not possible to confirm by genetic characterisation whether the strains present in the congenitally infected calves from Group A were NC-Argentina LP1 or the NC-1 strain because only one umbilical cord sample, *Neospora* DNA, was found (calf #6, see Table 3), and the DNA concentration in this sample was low. It is possible that the low detection of positive PCR samples in infected animals could be due to the existence of calf immunity or to the selected challenge strain. Benavides et al. [22] mentioned that in new-born calves, parasite DNA is sometimes difficult to detect, with histological lesions being rare, mild, and limited to brain tissues, which is probably due to the control of parasite multiplication by a competent foetal immune system that develops between mid to late gestation [7,32,33,43]. Additionally, although the NC-1 strain is considered a virulent strain in different works, several authors have reported that the prolonged in vitro passage of this isolate might attenuate its virulence [44,45]. Hence, it is possible that in the present study, the NC-1 strain could have attenuated virulence after serial passages in cell culture. If the challenge had been performed with a more pathogenic strain, perhaps more lesions and *Neospora*-positive DNA samples would have been found. At any rate, in the present study, vertical transmission could be corroborated by serology in calves before colostrum intake.

On the other hand, one abortion occurred in one Group A heifer at 35 days post challenge (#12), although *N. caninum* could not be confirmed as the cause of death. In addition to serological and molecular assays, histopathological examination was performed, and no compatible lesions were found. Interestingly, parasitaemia was detected in the dam at the moment of abortion. It is possible that placental and foetal immunocompetence play a role in avoiding parasite invasion [22]. However, no microscopic lesions were found in the placenta. In fact, parasitic DNA was not detected in foetal tissues from aborted foetuses. Unfortunately, the efficacy of this infection strategy to prevent abortion could not be evaluated in this study because the challenge was performed at Day 210 of gestation, and the main goal was to evaluate reactivation and vertical transmission. Considering that the aetiology of reproductive failure is multifactorial and that the experimental assays under field conditions cannot be completely controlled, the possible contribution of other non-infectious factors as the cause of abortion in this animal (nutritional, genetic, toxic, environmental, etc.) cannot be ruled out.

The protective immune response against *N. caninum* is dominated by the production of Th1 cytokines, although in pregnant cattle, such responses can be modulated to avoid the rejection or abortion of the foetus [7,20,41]. In the present study, notable changes in IL-10 levels were detected in Groups A (after challenge) and C at months 8 and 9 of gestation, thus evidencing a maternal immune response aimed at controlling the proinflammatory immune response. In addition, an increase in the IL-10 level in Group C was detected at month 7 of gestation, coinciding with the time of parasitic reactivation. IL-10 is a regulatory cytokine that is triggered when the Th1 response is exacerbated and, in this way, can downregulate proinflammatory responses [46]. On the other hand, significant changes in the TNF-α levels were only observed in Groups A and B after challenge, but the IFN-γ and IL-4 levels were not significantly modified, although a decreasing trend for both cytokines was observed, specifically after challenge and parasite reactivation. This fact could show a modulatory response that prevented an exacerbation of the Th1 response that could cause foetal or placental damage. This likely explains why no noticeable changes in IL-12 p40 levels were observed in any group beyond the significant increase observed in Group C prior to delivery. The absence of significant Th1 cytokine levels produced by PMBC agrees with previous reports that reported minor differences in the expression levels of these cytokines after experimental infection during the second and third trimesters of gestation [7,29,42]. Unfortunately, as the assay was developed in field conditions, the animals were only sampled monthly; hence, it cannot be ruled out that cytokine production might have occurred between the periods of monthly sampling dates.

## 5. Conclusions

The results of the present study show for the first time that the inoculation of live tachyzoites of *N. caninum* in prepubertal female calves is not effective in preventing the reactivation of the parasite during pregnancy, showing that the infected animals were unable to eliminate the parasite at their young age. In addition, although prepubertal infection elicited a specific immune response against *N. caninum* [11], this response was not sufficient to prevent congenital infection after a heterologous challenge. Therefore, we provide evidence that the use of live *N. caninum* tachyzoites in young animals as a strategy to induce protection is neither safe nor effective.

## Data Availability

Not applicable.

## References

[1] Lindsay D.S., Dubey J.P. (2020). Neosporosis, toxoplasmosis, and sarcocystosis in ruminants: An update. Vet. Clin. N. Am. Food Anim. Pract..

[2] Dubey J., Hemphill A., Calero-Bernal R., Schares G. (2017). Neosporosis in Animals.

[3] Reichel M.P., Ayanegui-Alcérreca M.A., Gondim L.F.P., Ellis J.T. (2013). What is the global economic impact of Neospora caninum in cattle—The billion dollar question. Int. J. Parasitol..

[4] Horcajo P., Regidor-Cerrillo J., Aguado-Martínez A., Hemphill A., Ortega-Mora L.M. (2016). Vaccines for bovine neosporosis: Current status and key aspects for development. Parasite Immunol..

[5] Staska L.M., McGuire T.C., Davies C.J., Lewin H.A., Baszler T.V. (2003). *Neospora caninum*-infected cattle develop parasite-specific CD4^+^ cytotoxic T lymphocytes. Infect. Immun..

[6] Almería S., Nogareda C., Santolaria P., Garcia-Ispierto I., Yániz J.L., López-Gatius F. (2009). Specific anti-*Neospora caninum* IgG1 and IgG2 antibody responses during gestation in naturally infected cattle and their relationship with gamma interferon production. Vet. Immunol. Immunopathol..

[7] Almería S., Serrano-Pérez B., López-Gatius F. (2017). Immune response in bovine neosporosis: Protection or contribution to the pathogenesis of abortion. Microb. Pathog..

[8] McAllister M.M., Björkman C., Anderson-Sprecher R., Rogers D.G. (2000). Evidence of point-source exposure to Neospora caninum and protective immunity in a herd of beef cows. J. Am. Vet. Med. Assoc..

[9] Innes E.A., Wright S.E., Maley S., Rae A., Schock A., Kirvar E., Bartley P., Hamilton C., Carey I.M., Buxton D. (2001). Protection against vertical transmission in bovine neosporosis. Int. J. Parasitol..

[10] Campero L.M., Venturini M.C., Moore D.P., Massola L., Lagomarsino H., García B., Bacigalupe D., Rambeaud M., Pardini L., Leunda M.R. (2015). Isolation and molecular characterization of a new *Neospora caninum* isolate from cattle in Argentina. Exp. Parasitol..

[11] Hecker Y.P., Regidor-Cerrillo J., Fiorani F., Horcajo P., Soria I., Gual I., Torioni S., Campero L.M., Echaide I.E., Álvarez-García G. (2019). Immune response to *Neospora caninum* live tachyzoites in prepubertal female calves. Parasitol. Res..

[12] Paré J., Thurmond M.C., Hietala S.K. (1996). Congenital *Neospora caninum* infection in dairy cattle and associated calfhood mortality. Can. J. Vet. Res..

[13] Dubey J.P., Hattel A.L., Lindsay D.S., Topper M.J. (1988). Neonatal *Neospora caninum* infection in dogs: Isolation of the causative agent and experimental transmission. J. Am. Vet. Med. Assoc..

[14] Hecker Y.P., Moore D.P., Quattrocchi V., Regidor-Cerrillo J., Verna A., Leunda M.R., Morrell E., Ortega-Mora L.M., Zamorano P., Venturini M.C. (2013). Immune response and protection provided by live tachyzoites and native antigens from the NC-6 Argentina strain of *Neospora caninum* in pregnant heifers. Vet. Parasitol..

[15] Rivera J.E.M., Hecker Y.P., Burucúa M.M., Cirone K.M., Cheuquepán F.A., Fiorani F., Dorsch M.A., Colque L.A., Cantón G.J., Marin M.S. (2021). Innate and humoral immune parameters at delivery in colostrum and calves from heifers experimentally infected with *Neospora caninum*. Mol. Immunol..

[16] Caspe S.G., Moore D.P., Leunda M.R., Cano D.B., Lischinsky L., Regidor-Cerrillo J., Álvarez-García G., Echaide I.G., Bacigalupe D., Ortega-Mora L.M. (2012). The *Neospora caninum*-Spain 7 isolate induces placental damage, fetal death and abortion in cattle when inoculated in early gestation. Vet. Parasitol..

[17] Campero L.M., Minkec L., Moré G., Rambeaud M., Bacigalupe D., Moore D.P., Hecker Y., Campero C.M., Schares G., Venturini M.C. (2015). Evaluation and comparison of serological methods for the detection of bovine neosporosis in Argentina. Rev. Argent. Microbiol..

[18] Buxton D., Maley S.W., Wright S., Thomson K.M., Rae A.G., Innes E.A. (1998). The pathogenesis of experimental neosporosis in pregnant sheep. J. Comp. Pathol..

[19] Campero C.M., Moore D.P., Odeón A.C., Cipolla A.L., Odriozola E. (2003). Aetiology of bovine abortion in Argentina. Vet. Res. Commun..

[20] Regidor-Cerrillo J., Arranz-Solís D., Benavides J., Gómez-Bautista M., Castro-Hermida J.A., Mezo M., Pérez V., Ortega-Mora L.M., González-Warleta M. (2014). *Neospora caninum* infection during early pregnancy in cattle: How the isolate influences infection dynamics, clinical outcome and peripheral and local immune responses. Vet. Res..

[21] Livak K.J., Schmittgen T.D. (2001). Analysis of Relative Gene Expression Data Using Real-Time Quantitative PCR and the 2^−ΔΔCT^. Methods.

[22] Benavides J., Collantes-Fernández E., Ferre I., Pérez V., Campero C., Mota R., Innes E., Ortega-Mora L.M. (2014). Experimental ruminant models for bovine neosporosis: What is known and what is needed. Parasitology.

[23] Williams D.J., Guy C.S., Smith R.F., Guy F., McGarry J.W., McKay J.S., Trees A.J. (2003). First demonstration of protective immunity against foetopathy in cattle with latent *Neospora caninum* infection. Int. J. Parasitol..

[24] De Marez T., Liddell S., Dubey J.P., Jenkins M.C., Gasbarre L. (1999). Oral infection of calves with *Neospora caninum* oocysts from dogs: Humoral and cellular immune responses. Int. J. Parasitol..

[25] Maley S.W., Buxton D., Thomson K.M., Schriefer C.E.S., Innes E.A. (2001). Serological analysis of calves experimentally infected with *Neospora caninum*: A 1-year study. Vet. Parasitol..

[26] Klevar S., Kulberg S., Boysen P., Storset A.K., Moldal T., Björkman C., Olsen I. (2007). Natural killer cells act as early responders in an experimental infection with *Neospora caninum* in calves. Int. J. Parasitol..

[27] Quintanilla-Gozalo A., Pereira-Bueno J., Tabarés E., Innes E.A., González-Paniello R., Ortega-Mora L.M. (1999). Seroprevalence of *Neospora caninum* infection in dairy and beef cattle in Spain. Int. J. Parasitol..

[28] Maley S.W., Buxton D., Rae A.G., Wright S.E., Schock A., Bartley P.M., Esteban-Redondo I., Swales C., Hamilton C.M., Sales J. (2003). The pathogenesis of neosporosis in pregnant cattle: Inoculation at mid-gestation. J. Comp. Pathol..

[29] Williams D.J., Guy C.S., McGarry J.W., Guy F., Tasker L., Smith R.F., MacEachern K., Cripps P.J., Kelly D.F., Trees A.J. (2000). Neospora caninum-associated abortion in cattle: The time of experimentally-induced parasitaemia during gestation determines foetal survival. Parasitology.

[30] Guy C.S., Williams D.J.L., Kelly D.F., McGarry J.W., Guy F., Björkman C., Smith R.F., Trees A.J. (2001). *Neospora caninum* in persistently infected, pregnant cows: Spontaneous transplacental infection is associated with an acute increase in maternal antibody. Vet. Rec..

[31] Nogareda C., López-Gatius F., Santolaria P., García-Ispierto I., Bech-Sàbat G., Pabón M., Mezo M., Gonzalez-Warleta M., Castro-Hermida J.A., Yániz J. (2007). Dynamics of anti-*Neospora caninum* antibodies during gestation in chronically infected dairy cows. Vet. Parasitol..

[32] Williams D.J., Guy C.S., Smith R.F., Ellis J., Björkman C., Reichel M.P., Trees A.J. (2007). Immunization of cattle with live tachyzoites of *Neospora caninum* confers protection against fetal death. Infect. Immun..

[33] Rojo-Montejo S., Collantes-Fernández E., Blanco-Murcia J., Rodríguez-Bertos A., Risco-Castillo V., Ortega-Mora L. (2009). Experimental infection with a low virulence isolate of *Neospora caninum* at 70 days gestation in cattle did not result in foetopathy. Vet. Res..

[34] Rojo-Montejo S., Collantes-Fernandez E., Perez-Zaballos F., Rodriguez-Marcos S., Blanco-Murcia J., Rodriguez-Bertos A., Prenafeta A., Ortega-Mora L.M. (2013). Effect of vaccination of cattle with the low virulence Nc-Spain 1H isolate of *Neospora caninum* against a heterologous challenge in early and mid-gestation. Vet. Res..

[35] Weber F.H., Jackson J.A., Sobecki B., Choromansky L., Olsen M., Meinert T., Frank R., Reichel M.P., Ellis J.T. (2013). On the efficacy and safety of vaccination with live tachyzoites of *N. caninum* for prevention of *Neospora*-associated fetal loss in cattle. Clin. Vaccine Immunol..

[36] Mazuz M.L., Fisha L., Wolkomirsky R., Leibovich B., Reznikov D., Savitsky I., Golenser J., Shkap V. (2015). The effect of a live *Neospora caninum* tachyzoite vaccine in naturally infected pregnant dairy cows. Prev. Med..

[37] Mazuz M.L., Leibovich B., Savitsky I., Blinder E., Yasur-Landau D., Lavon Y., Sharir B., Tirosh-Levy S. (2021). The effect of vaccination with *Neospora caninum* live-frozen tachyzoites on abortion rates of naturally infected pregnant cows. Vaccines.

[38] Regidor-Cerrillo J., Gómez-Bautista M., Pereira-Bueno J., Aduriz G., Navarro-Lozano V., Risco-Castillo V., Férnandez-García A., Pedraza-Díaz S., Ortega-Mora L.M. (2008). Isolation and genetic characterization of *Neospora caninum* from asymptomatic calves in Spain. Parasitology.

[39] Benavides J., Katzer F., Maley S.W., Bartley P.M., Canton G., Palarea-Albaladejo J., Purslow C.A., Pang Y., Rocchi M.S., Chianini F. (2012). High rate of transplacental infection and transmission of Neospora caninum following experimental challenge of cattle at day 210 of gestation. Vet. Res..

[40] Rosbottom A., Guy C.S., Gibney E.H., Smith R.F., Valarcher J.F., Taylor G., Williams D.J.L. (2007). Peripheral immune responses in pregnant cattle following *Neospora caninum* infection. Parasite Immunol..

[41] Rosbottom A., Gibney H., Kaiser P., Hartley C., Smith R.F., Robinson R., Kipar A., Williams D.J. (2011). Up regulation of the maternal immune response in the placenta of cattle naturally infected with *Neospora caninum*. PLoS ONE.

[42] Gibney E.H., Kipar A., Rosbottom A., Guy C.S., Smith R.F., Hetzel U., Trees A.J., Williams D.J. (2008). The extent of parasite-associated necrosis in the placenta and foetal tissues of cattle following Neospora caninum infection in early and late gestation correlates with foetal death. Int. J. Parasitol..

[43] Bartley P.M., Katzer F., Rocchi M.S., Maley S.W., Benavides J., Nath M., Pang Y., Canton G., Thomson J., Chianini F. (2013). Development of maternal and foetal immune responses in cattle following experimental challenge with *Neospora caninum* at day 210 of gestation. Vet. Res..

[44] Bartley P.M., Wright S., Chianini F., Buxton D., Innes E.A. (2008). Inoculation of Balb/c mice with live attenuated tachyzoites protects against a lethal challenge of *Neospora caninum*. Parasitology.

[45] Aminia L., Namavarib M., Khodakaram-Taftia A., Divarc M.R., Hosseinib S.M.H. (2020). The evaluation of attenuated *Neospora caninum* by long-term passages on murine macrophage cell line in prevention of vertical transmission in mice. Vet. Parasitol..

[46] Bancherau J., Pascual V., O’Garra A. (2012). From IL-2 to IL-37: The expanding spectrum of anti-inflammatory cytokines. Nat. Immunol..

